# Injectable platelet-rich fibrin for corneal epithelium healing: An in vivo confocal microscopy study after crosslinking

**DOI:** 10.5935/0004-2749.2024-0326

**Published:** 2025-06-24

**Authors:** Alperen Bahar, Huri Sabur, Mutlu Acar

**Affiliations:** 1 Department of Ophthalmology, Diskapi Yildirim Beyazit Training and Research Hospital, Ankara, Turkey; 2 Department of Ophthalmology, Yuksek Ihtisas University, Ankara, Turkey

**Keywords:** Keratoconus, Platelet-rich fibrin, Epithelium, cor-neal, Corneal crosslinking, Wound healing

## Abstract

**Purpose:**

This study was conducted to investigate the effect of injectable platelet-rich fibrin on the recovery of compromised epithelium due to crosslinking treatment.

**Methods:**

In this comparative study, the epithelial closure rates and in vivo confocal biomicroscopy results of 26 patients with keratoconus who underwent subconjunctival injection of injectable platelet-rich fibrin near the limbus after epithelium-off corneal crosslinking treatment were compared with those of 25 patients who did not receive the injection of injectable platelet-rich fibrin.

**Results:**

The average time to epithelial defect closure in the injectable platelet-rich fibrin group was 2.76 ± 0.90 days compared to 3.56 ± 0.86 days in the non-injectable platelet-rich fibrin group (p=0.003). At the end of the 1st month, the mean subbasal nerve plexus density was 1.26 ± 1.61 nerves/mm^2^ in the injectable platelet-rich fibrin group, whereas it was 0.72 ± 0.89 nerves/mm^2^ in the non-injectable platelet-rich fibrin group (p=0.016). By the 3rd month, the density increased to 3.42 ± 1.13 nerves/mm^2^ in the injectable platelet-rich fibrin group and 2.36 ± 1.15 nerves/mm^2^ in the non-injectable platelet-rich fibrin group (p=0.002). Similarly, the anterior stromal keratocyte density at the end of the 1st month was 93.6 ± 33.5 cells/mm^2^ in the injectable platelet-rich fibrin group compared to 67.3 ± 26.4 cells/mm^2^ in the non-injectable platelet-rich fibrin group (p=0.001). By the end of the 3rd month, the density increased to 255.2 ± 45.7 cells/mm^2^ in the injectable platelet-rich fibrin group and 222.1 ± 43.6 cells/mm^2^ in the non-injectable platelet-rich fibrin group (p=0.011). In the non-injectable platelet-rich fibrin group, one patient developed a sterile infiltrate at the end of the 1st week, whereas no complications were observed in the injectable platelet--rich fibrin group.

**Conclusion:**

Subconjunctival injectable platelet-rich fibrin application is an effective and safe method for corneal epithelial healing after crosslinking treatment.

## INTRODUCTION

Since its first report in 1984, the use of autologous platelet-rich plasma (PRP) has gained considerable popularity and widespread acceptance as a valuable treatment option for various ocular surface disorders, including corneal ulcers, chemical injury, and limbal stem cell deficiency^(1)^. Moreover, studies have demonstrated its efficacy in improving the healing of epithelial defects after procedures such as corneal crosslinking (CXL), penetrating and lamellar keratoplasty, and photorefractive keratectomy^(2^-^5)^.

The basis of PRP treatments lies in platelet-derived growth factors and their positive effects on healing. Nevertheless, in PRP treatment, the fibrin structure, which is crucial for tissue healing and serves as a site for platelets to settle and secrete growth factors, is absent because anticoagulants prevent the conversion of fibrinogen into fibrin^(6)^.

In 2001, Choukroun et al. described platelet-rich fibrin (PRF), a three-dimensional polymerized autologous fibrin matrix enriched with platelets and their biologically active components^(7)^. PRF consists of a platelet-containing fibrin structure enriched as a result of natural coagulation in tubes that do not contain anticoagulants. Platelets activated within the fibrin structure release growth factors for an extended period of time^(6)^. Furthermore, anticoagulants are prevented from reducing the effectiveness of growth factors^(7)^. In 2017, Choukroun et al. described a PRF format (injectable PRF) that can be injected into tissues by modifying the centrifuge settings and the characteristics of the tube used for blood collection^(8)^. This format allows for liquid injection over a 15- to 20-min period, and fibrin formation occurs at the injection site.

Epithelium-off (epi-off) corneal CXL procedures involve the removal of the corneal epithelium to improve the penetration of riboflavin into the tissue. However, this process has risks such as corneal haze, infection, and severe pain. Persistent epithelial defects and even corneal melting have been reported as potential outcomes^(9)^. Therefore, it is crucial for the epithelium to heal rapidly, for which various treatments such as the application of artificial tears are utilized.

Despite the increasing evidence supporting the regenerative potential of PRF, no studies have explored its use for corneal epithelial healing and nerve regeneration. Considering that limbal stem cells, essential for corneal repair, migrate toward the central cornea upon epithelial injury^(10)^, targeted i-PRF injections near the limbus may improve the process of regeneration. Previous research has demonstrated the positive effects of i-PRF on wound healing in other tissues^(11)^; however, its potential for accelerating corneal epithelial closure and nerve recovery remains unexplored.

Therefore, this study was conducted to address this gap by investigating the effects of i-PRF on epithelial healing and the findings of in vivo confocal microscopy (IVCM) after corneal CXL. Faster epithelial recovery and improved nerve regeneration could provide significant clinical benefits, including reduced patient discomfort, shorter recovery times, fewer complications, improved visual rehabilitation, and potential economic advantages. By introducing i-PRF as a novel therapeutic approach in corneal healing, this study provides valuable insights into its regenerative potential and clinical applicability.

## METHODS

This retrospective and comparative study evaluated the data of 51 patients who underwent epi-off CXL for keratoconus between 2021 and 2023. Written informed consent was obtained from all patients, and this study was conducted according to the Declaration of Helsinki with approval obtained from the Etlik City Hospital Ethics Committee of the University of Health Sciences (AESH-EK1-2023-653).

### Study population

Of the 51 patients who underwent CXL treatment for progressive keratoconus, 26 underwent subconjunctival i-PRF immediately after the procedure. Patients’ age was 15-40 years. Patients with additional conditions that could impair wound healing, such as diabetes mellitus, autoimmune diseases, and collagen tissue disorders, were excluded. Moreover, patients who were nonadherent to the specified follow-ups, had incomplete data and other eye diseases, were undergoing concurrent treatments, or were using contact lenses were excluded. Keratoconus progression was evaluated based on either an increase of ≥1.00 D in the steepest keratometry (K) measurement or a loss of at least two lines of best spectacle-corrected visual acuity within the past 12 months.

### Surgical technique

After applying topical anesthesia with 0.5% proparacaine drops (Alcaine, Alcon-Couvreur, Belgium) and a 30-s application of 20% alcohol, 8 mm of the central corneal epithelium was gently scraped using a blunt spatula. Then, riboflavin, 0.1% riboflavin with saline, and hydroxypropyl methylcellulose drops (VibeXRapid, Avedro, Waltham, MA, USA) were applied to the cornea at 2-min intervals for 10 min. After the instillation of riboflavin, UV-A light (Avedro KXL, Avedro Inc., USA) was applied at an intensity of 9 mW/cm^2^ for 10 min (cumulative dose: 5.4 J/cm^2^). Throughout the 10-min irradiation period, riboflavin instillation was continued every 2 min. At the end of this stage, the corneal surface was rinsed with sterile balanced salt solution, a soft bandage contact lens (Air Optix Night & Day AQUA, Alcon, USA) was placed over the cornea, and one drop of ocular moxifloxacin 0.5% (Vigamox; Alcon Laboratories Inc., USA) was administered.

### Preparation and application of i-PRF

**Figure 1:**
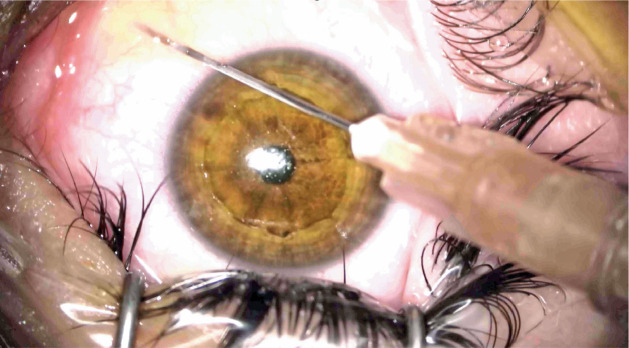
i-PRF administered from four quadrants close to the limbus.

After the completion of CXL treatment, 10 ml of blood was collected from the patient’s antecubital vein into i-PRF tubes (Z No Additive Tube, BD Vacutainer). Immediately after collection, the blood was centrifuged at 700 rpm for 3 min using a table centrifuge system (DLAB 0506, DLAB SCIENTIFIC CO., LTD., Beijing, China) in the operating room. Patients in the i-PRF group received subconjunctival injections of 0.1 ml of i-PRF close to the limbus, administered from four quadrants (Figure 1).

After surgery, the patients were administered topical moxifloxacin 0.5% (Vigamox; Alcon Laboratories, Inc.), loteprednol etabonate ophthalmic suspension 0.5% (Lotemax; Bausch & Lomb) four times daily, and artificial tears (EYESTIL, 0.15% Sodium Hyaluronate, SIFI, Italy) every 3 h until epithelial healing was complete. Then, the bandage contact lens was removed, and moxifloxacin was discontinued; however, loteprednol etabonate ophthalmic suspension 0.5% (Lotemax; Bausch & Lomb) was continued four times daily for 1 month.

### Patient evaluation and follow-up examinations

Corneal topographic indices, including flat keratometry (K1), steep keratometry (K2), average keratometry (K Avg), and central corneal thickness, were evaluated using Placido disk topography with Scheimpflug tomography of the anterior segment (Sirius; Costruzione Strumenti Oftalmici, Florence, Italy) by the same trained examiner.

After the CXL procedure, the size of the corneal epithelial defect was promptly recorded using a digital camera (DC-4 Grabber, Topcon, Japan) attached to a slit-lamp biomicroscope. To conduct this procedure, a drop of proparacaine (proparacaine hydrochloride 0.5%, Alcaine, Alcon, USA) was administered, followed by placing a sterile fluorescein strip moistened with saline solution in the lower conjunctival sac. The patient was then instructed to blink several times, after which three photographs were taken under cobalt-blue filtered light with a camera magnification of 1x and a slit-lamp magnification of 10x. Then, a silicone hydrogel bandage contact lens (Lotrafilcon A, Air Optix Night & Day, Alcon, USA) was applied to the patient’s eye. This contact lens was replaced daily under slit-lamp examination for photographic documentation until complete reepithelialization was achieved.

Postoperative follow-up examinations were conducted at 1, 3, and 6 months, involving ultrastructural analysis by IVCM (HRT III Cornea Module; Rostock, Heidelberg, Germany).

Any adverse events during each postoperative visit were monitored, with particular focus on infection, inflammation, and any other complications. Any obser-ved complications were documented and managed accordingly.

For IVCM evaluations, all examinations were conducted by a single experienced examiner (HS). Section and volume scans of the central cornea were recorded using the Heidelberg HRT-III microscope, with a resolution of 384 × 384 pixels and a field of view of 400 × 400 µm^2^. To ensure consistency, the IVCM settings were standardized for all evaluations. Three high-quality images (without motion artifacts) of the basal epithelium, posterior stroma, and subbasal nerve plexus were selected and analyzed by the examiner (HS), who followed a detailed protocol for image acquisition and analysis. The examiner was blinded to the treatment group during the evaluations to minimize potential observer bias. The average value of these three measurements was used for further comparative analysis. The basal epithelium was defined as the first three clear scans anterior to Bowman’s layer, and the anterior stroma was defined as the first three clear images immediately posterior to Bowman’s layer. For cell counting, the instrument-based software provided semiautomated cell density measurements. The cells were manually marked, and the software automatically calculated the cell density (cells/mm^2^).

### Image analysis and statistics

The captured images were analyzed using the ImageJ software (NIH, Bethesda, MD, USA). The horizontal corneal diameter was used to set the scale in millimeters. Two ophthalmologists, blinded to the patient groups, manually outlined the margins of the epithelial defects. Then, the size of the epithelial defect was calculated by converting the pixel values of the selected defect area into square millimeters. The size of the defects was evaluated initially (immediately after the CXL procedure) and throughout the postoperative daily visits until complete reepithelialization was achieved. The dimensions of the epithelial defect and the number of days when complete epithelialization was achieved were recorded.

Statistical analyses were conducted using the Statistical Package for the Social Sciences version 21.0 software for Windows (SPSS Inc, Chicago, IL) after transferring the data to the computer. Power analysis was conducted using the GPower 3.1 software. Based on the study by Sabur et al.^(12)^, which compared preoperative subbasal nerve plexus density (10.73 ± 3.52) with 6-month postoperative subbasal nerve plexus density (7.02 ± 1.42) after corneal CXL, an effect size of 1.209 was calculated. With an alpha of 0.05 and a power of 0.95, the analysis indicated that at least 10 patients per group would be required to achieve statistically significant results using the Wilcoxon signed-rank test for paired ordinal data. The normality of data was analyzed using the Shapiro-Wilk test. The chi-square test was used to compare categorical data between two groups, and an independent samples t-test and Mann-Whitney U test were used for comparing continuous data. P<0.05 was considered statistically significant.

## RESULTS

Among the 51 patients who underwent corneal collagen CXL treatment, no significant differences were observed in the preoperative corneal thickness and keratometry values between the group of 26 patients who received i-PRF and the group that did not. Table 1 shows the baseline characteristics of the patients.

**Table 1:** Preoperative characteristics of the study groups

Mean ± SD n (%)	i-PRF-applied group (n=26)	i-PRF-nonapplied group (n=25)	p-value
Age	23.42 ± 3.73	21.84 ± 2.83	0.58^*^
Sex			0.33^**^
Male	13 (25.4%)	14 (27.4%)	
Female	13 (25.4%)	11 (21.6%)	
Flat keratometry (K1, D)	48.06 ± 1.95	48.02 ± 1.49	0.23^*^
Steep keratometry (K2, D)	52.04 ± 3.38	51.25 ± 1,87	0.16^*^
Average keratometry (K Avg, D)	50.06 ± 2.26	49.64 ±1.62	0.74^*^
Central corneal thickness (µm)	475.03 ± 28.07	469.44 ± 32.75	0.45^*^

* Independent sample t-test;

** chi-square test.

**Figure 2:**
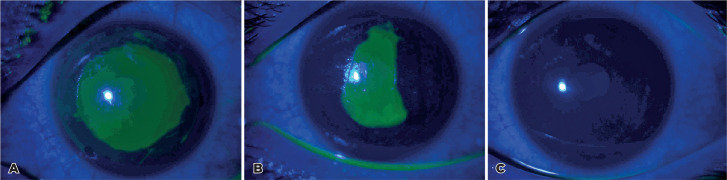
Slit-lamp images of the fluorescein-stained epithelial defect in the i-PRF group: (A) Immediately after surgery, (B) postoperative day 1, and (C) postoperative day 2.

**Figure 3:**
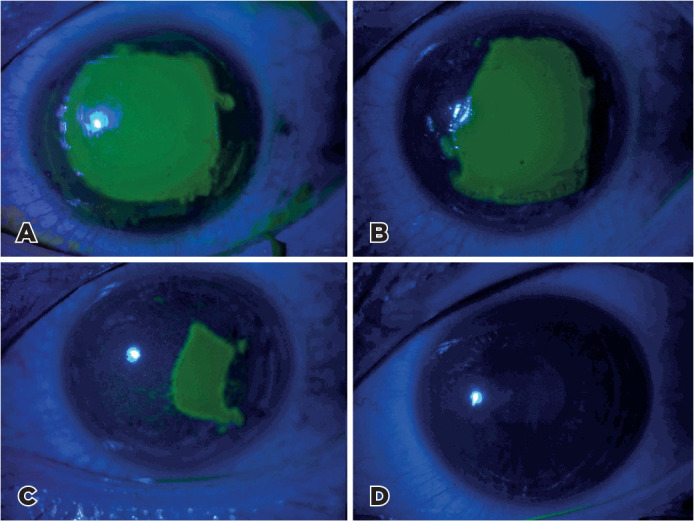
Slit-lamp images of the fluorescein-stained epithelial defect in the non-i-PRF group: (A) Immediately after surgery, (B) postoperative day 1, (C) postoperative day 2, and (D) postoperative day 3.

A comparison of the daily epithelial healing rates between the groups showed that the epithelial defect closed significantly faster after i-PRF. In the i-PRF group, the average duration for epithelial defect closure was 2.76 ± 0.90 days, whereas in the non-i-PRF group, it was 3.56 ± 0.86 days (p=0.003) (Table 2). In the non-i-PRF group, one patient developed a sterile infiltrate at the end of the 1st week, which was resolved with hourly topical steroid treatment (0.1% dexamethasone, MAXIDEX, ALCON Couvreur N.V, Belgium), whereas no complications developed in the i-PRF group. Changes in epithelial defect size are shown in Figures 2 and 3.

**Table 2:** Comparison of epithelial defect size and time to complete closure between the groups

	i-PRF-applied group (n=26) mean ± SD	i-PRF-nonapplied group (n=25) mean ± SD	p-value
Epithelial defect size (mm^2^)			
Postoperative immediate	47.93 ± 0.41	47.91 ± 0.73	0.90^*^
Postoperative day 1	20.40 ± 6.45	25.42 ± 4.02	**0.002** ^*^
Postoperative day 2	6.03 ± 2.57	8.60 ± 2.48	**0.001** ^*^
Postoperative day 3	0.83 ± 0.96	1.73 ± 1.07	**0.003** ^**^
Time to closure (days)	2.76 ± 0.90	3.56 ± 0.86	**0.003** ^**^
Postoperative sterile infiltrate	0	1	0.49^***^

* Independent sample t-test;

** Mann-Whitney U test;

*** Chi-square test.

Bold type: statistically significant.

**Figure 4:**
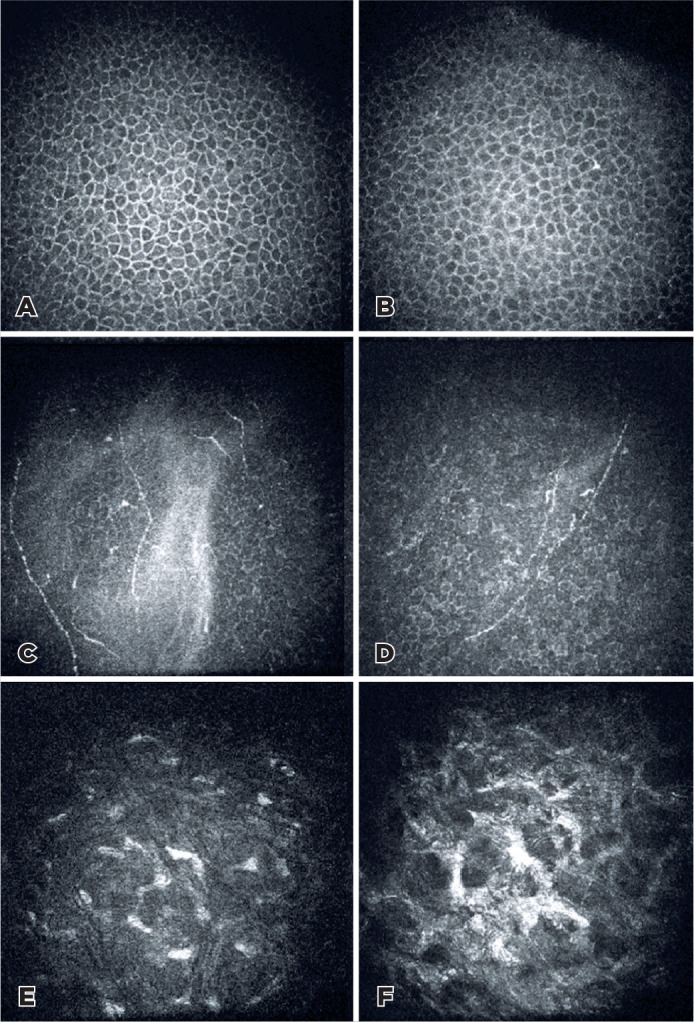
IVCM images of basal epithelial cells (A,B), subbasal nerve plexus (C,D), and anterior stromal cells (E,F) in the i-PRF (A,C,E) and non-i-PRF (B,D,F) groups at the 3rd month after CXL.

In both groups, the mean basal epithelial cell density, subbasal nerve plexus density, and anterior stromal cell density significantly decreased compared with baseline values at all postoperative visits (Table 3 and Figure 4).

**Table 3:** IVCM findings of i-PRF and non-i-PRF groups before CXL and at the 1st, 3rd, and 6th month

	i-PRF-applied group (n=26) mean ± SD	i-PRF-nonapplied group (n=25) mean ± SD	p-value
Basal epithelial cell density (cells/mm^2^)			
Preoperative	4321 ± 209	4356 ± 198	0.55^*^
Postoperative 1 month	3837 ± 203	3671 ± 201	**0.005** ^*^
Postoperative 3 months	4027 ± 185	3899 ± 158	**0.011** ^*^
Postoperative 6 months	4184 ± 188	4097 ± 158	0.08^*^
Subbasal nerve plexus density (nerve/mm^2^)			
Preoperative	11.07 ± 1.71	10.92 ± 1.75	0.73^**^
Postoperative 1 month	1.26 ± 0.61	0.72 ± 0.89	0.016^**^
Postoperative 3 months	3.42 ± 1.13	2.36 ± 1.15	0.002^**^
Postoperative 6 months	7.19 ± 1.60	6.36 ± 1.41	0.055^**^
Anterior stromal cell density (cells/mm^2^)			
Preoperative	553.2 ± 99.5	589.5 ± 79.8	0.43^*^
Postoperative 1 month	93.6 ± 33.5	67.3 ± 26.4	0.001^*^
Postoperative 3 months	255.2 ± 45.7	222.1 ± 43.6	**0.011** ^*^
Postoperative 6 months	350.7 ± 60.9	328.5 ± 50.7	0.16^*^

* Independent sample t-test;

** Mann-Whitney U test.

Bold type: statistically significant.

Because epithelial healing progressed more rapidly in the i-PRF group, the basal epithelial cell density was significantly higher in this group than in the non-i-PRF group at the 1st and 3rd postoperative months. Meanwhile, the IVCM findings revealed comparable basal epithelial cell density between the groups at the 6-month postoperative period (Table 3).

At 1 month, subbasal nerves were absent in 61.5% and 72% of the eyes in the i-PRF and non-i-PRF groups, respectively; at 3 months, these rates respectively decreased to 30.76% and 40%. Subbasal nerve plexus densities were significantly higher in the i-PRF group at 1 and 3 months postoperatively; however, at 6 months, although the subbasal nerve plexus density remained higher in both groups, there was no statistically significant difference between them (Table 3).

Anterior stromal edema with a honeycomb-like appearance was detected in all eyes postoperatively at the 1-month time point, which continued in the non-i-PRF group at 3 months. The mean anterior stromal keratocyte densities in the i-PRF group were significantly higher than those in the non-i-PRF group at both 1 and 3 months postoperatively. However, at 6 months, despite higher keratocyte densities in the i-PRF group, there was no statistically significant difference between the two groups (Table 3).

## DISCUSSION

Epi-off CXL procedures, involving the removal of the corneal epithelium to improve riboflavin penetration into the corneal stroma, are widely considered the most effective method for treating keratoconus and other corneal ectatic disorders^(13)^. Nevertheless, the removal of the epithelium has inherent risks, including complications such as infectious keratitis, haze, sterile infiltrate, diffuse lamellar keratitis, and significant pain^(14)^, which persist until the complete healing of the epithelial defect. Therefore, various agents have been investigated for facilitating faster and appropriate healing of the epithelium, including tear drops containing sodium hyaluronate and trehalose, the regenerating agent Cacicol^®^-RGTA, dexpanthenol, and omega-3 fatty acids^(12^,^15^-^18)^. Another agent used for this purpose is platelet-rich blood products. By accelerating recovery after ocular surface diseases and epithelial damage, they have demonstrated efficacy in numerous studies and have become a standard component of daily practice^(19)^. Their effective uses, which originated with autologous serum, have expanded with new-generation blood products such as PRP, solid PRP, PRF, i-PRF, and platelet-rich growth factor (PRGF), all of which are obtained through various centrifuge protocols. The present study, conducted by considering this information, demonstrated that subconjunctivally applied i-PRF accelerated epithelial renewal.

The results of studies conducted using autologous serum have indicated that it accelerates the closure of epithelial defects because of the presence of epithelial growth factors in it. For instance, Chen et al. demonstrated the efficacy of autologous serum in promoting graft reepithelialization after penetrating keratoplasty, particularly among patients with diabetes and larger grafts^(3)^. Moreover, Akcam et al. demonstrated that autologous serum accelerates epithelial healing after photorefractive keratectomy, and Kirgiz et al. also reported a similar effect after CXL treatment^(5^,^20)^. Recent studies have also reported similar findings when PRP was used directly instead of autologous serum. For instance, Kamiya et al. found that epithelial healing was faster in patients using PRP drops after phototherapeutic keratectomy^(21)^. Similarly, Okumura et al. showed that PRP stored at 4°C for 4 weeks promoted faster epithelial healing than autologous serum^(22)^. Furthermore, Anitua et al. compared PRGF drops with insulin and autologous serum and found that PRGF accelerated the biological activity and proliferation processes of ocular surface cells compared with other treatments^(23)^. In addition, PRGF further reduced the levels of fibrosis markers. Marquez De Aracena et al. also demonstrated the beneficial effects of subconjunctival PRP treatment in patients with ocular alkali burn injury^(24)^. Moreover, Tanidir et al. reported that subconjunctival PRP treatment accelerated corneal epithelial healing in rabbits^(25)^.

Consistent with previous studies, our findings suggest that i-PRF accelerates epithelial healing. Nevertheless, in our study, i-PRF was administered via the subconjunctival route, which provides several advantages over topical drop administration. By bypassing the corneal epithelial barrier, it allows for better tissue penetration and undergoes fibrin transformation at the injection site, enabling prolonged release and less frequent administration. Nonetheless, it also has the inherent risks of an invasive procedure and may cause negative patient perceptions due to pain and anxiety.

IVCM is a powerful technique for examining corneal microstructural changes in vivo, allowing detailed observation of CXL-induced effects. After epi-off CXL, immediate subbasal nerve loss and keratocyte apoptosis occur due to epithelial debridement and UV-A-induced photonecrosis, as reported in previous studies^(26^-^29)^. In epi-off CXL alone, subbasal nerve regeneration and keratocyte repopulation are typically complete within 12 months^(26^-^28)^. Nevertheless, Alessio^(29)^ reported delayed keratocyte repopulation for up to 4 years in CXL plus PRK, attributing this to the disruption of Bowman’s layer and deeper penetration of riboflavin, which cause stromal compactness and stiffness. Our study investigated whether i-PRF accelerates keratocyte recovery in the first 6 months. Anterior stromal edema with a honeycomb-like pattern was detected in both groups but persisted up to 3 months postoperatively in the non-iPRF group (Figure 4). In addition, hyper-reflective needle-shaped microbands and fragmented keratocyte nuclei were detected in both groups, although keratocyte repopulation was more obvious in the i-PRF group. This may be due to the promotion of faster epithelial healing by i-PRF, its growth factor content, and anti-inflammatory effects. However, because our study reports only 6-month outcomes, long-term effects beyond 12 months remain unknown.

In a previous study, we demonstrated the effectiveness of i-PRF on epithelial and autograft healing after pterygium surgery^(30)^. Although i-PRF appears similar to PRP, it has important differences. i-PRF does not contain anticoagulants and additives. Platelets are activated naturally, and long-term platelet-derived growth factors are released in the fibrin matrix formed. It has been demonstrated that platelets within the fibrin matrix formed within 15 min after injection continue to release growth factors for up to 10 days^(31)^.

In terms of complications, none of the patients in either group developed corneal haze that could threaten their vision. Peripheral sterile corneal infiltrate developed in one patient in the non-i-PRF group, which resolved with steroid treatment. In a previous study of a series of 459 patients who underwent epi-off CXL treatment, a sterile corneal infiltrate was detected in 19 patients^(32)^. This result in both groups is consistent with the literature. Corneal sterile infiltrates result from inflammation due to debridement detected after epi-off CXL. One of the reasons for the hesitation concerning the use of blood products in healing is the presence of white blood cells in them. The potential of these cells to increase inflammation has been questioned. In contrast to this view, there is also evidence on the anti-inflammatory activity of i-PRF at the injection site^(33)^. In our study, no inflammatory reaction occurred at the injection site or the cornea.

There were several limitations in this study. First, a relatively small number of patients were investigated with no control group other than i-PRF, which could have included the use of autologous serum or other platelet-rich blood products. Moreover, the amount of i-PRF applied subconjunctivally was based on previous subconjunctival PRP applications, emphasizing the need for an appropriate dose study. Despite the stringent rules of the study for patient follow-up and data validity, its retrospective nature remains a limiting factor. Future studies with a larger cohort would be valuable for further validating the findings and establishing a stronger correlation between i-PRF administration and corneal healing. Furthermore, our study was conducted on healthy eyes without any conditions that could slow down epithelial healing. The effects of i-PRF in diseases that impede epithelial healing, such as diabetes mellitus, or in the treatment of ulcers with challenging epithelial defects, such as metaherpetic ulcers, may be the subject of future research. Another limitation is the absence of patient-reported outcomes (PROs), such as pain scores, visual recovery, and quality-of-life evaluations. As this is a retrospective study, these PROs could not be incorporated, which may limit a comprehensive evaluation of the benefits of i-PRF on patients’ subjective experiences and overall well-being. Future prospective studies could address this limitation by including these outcomes. In addition, the follow-up duration in this study was limited to 6 months, which may not completely capture long-term outcomes, including sustained nerve regeneration or the potential recurrence of epithelial defects. A longer follow-up duration would provide valuable insights into these aspects and allow for a more complete understanding of the long-term efficacy and safety of i-PRF.

In conclusion, subconjunctival i-PRF was effective and safe for corneal epithelial healing after CXL treatment in patients with keratoconus. This novel application also promoted faster corneal reepithelialization, nerve regeneration, and keratocyte repopulation. However, prospective studies with longer follow-up periods and a larger number of patients are necessary to further support our findings.

## Data Availability

The datasets generated during and/or analyzed during the current study are available in the https://doi.org/10.7910/DVN/ESKFGO
